# Pro-Arrhythmic Signaling of Thyroid Hormones and Its Relevance in Subclinical Hyperthyroidism

**DOI:** 10.3390/ijms21082844

**Published:** 2020-04-19

**Authors:** Narcis Tribulova, Lin Hai Kurahara, Peter Hlivak, Katsuya Hirano, Barbara Szeiffova Bacova

**Affiliations:** 1 Centre of Experimental Medicine, Slovak Academy of Sciences, Institute for Heart Research, 84104 Bratislava, Slovakia; 2 Department of Cardiovascular Physiology, Faculty of Medicine, Kagawa University, Kagawa 76 0793, Japan; hailin@med.kagawa-u.ac.jp (L.H.K.); khirano@med.kagawa-u.ac.jp (K.H.); 3 Department of Arrhythmias and Pacing, National Institute of Cardiovascular Diseases, Pod Krásnou Hôrkou 1, 83348 Bratislava, Slovakia; hlivakp@gmail.com

**Keywords:** thyroid diseases, thyroid hormone signaling, cardiac arrhythmias

## Abstract

A perennial task is to prevent the occurrence and/or recurrence of most frequent or life-threatening cardiac arrhythmias such as atrial fibrillation (AF) and ventricular fibrillation (VF). VF may be lethal in cases without an implantable cardioverter defibrillator or with failure of this device. Incidences of AF, even the asymptomatic ones, jeopardize the patient’s life due to its complication, notably the high risk of embolic stroke. Therefore, there has been a growing interest in subclinical AF screening and searching for novel electrophysiological and molecular markers. Considering the worldwide increase in cases of thyroid dysfunction and diseases, including thyroid carcinoma, we aimed to explore the implication of thyroid hormones in pro-arrhythmic signaling in the pathophysiological setting. The present review provides updated information about the impact of altered thyroid status on both the occurrence and recurrence of cardiac arrhythmias, predominantly AF. Moreover, it emphasizes the importance of both thyroid status monitoring and AF screening in the general population, as well as in patients with thyroid dysfunction and malignancies. Real-world data on early AF identification in relation to thyroid function are scarce. Even though symptomatic AF is rare in patients with thyroid malignancies, who are under thyroid suppressive therapy, clinicians should be aware of potential interaction with asymptomatic AF. It may prevent adverse consequences and improve the quality of life. This issue may be challenging for an updated registry of AF in clinical practice. Thyroid hormones should be considered a biomarker for cardiac arrhythmias screening and their tailored management because of their multifaceted cellular actions.

## 1. Introduction

**Figure 1:**
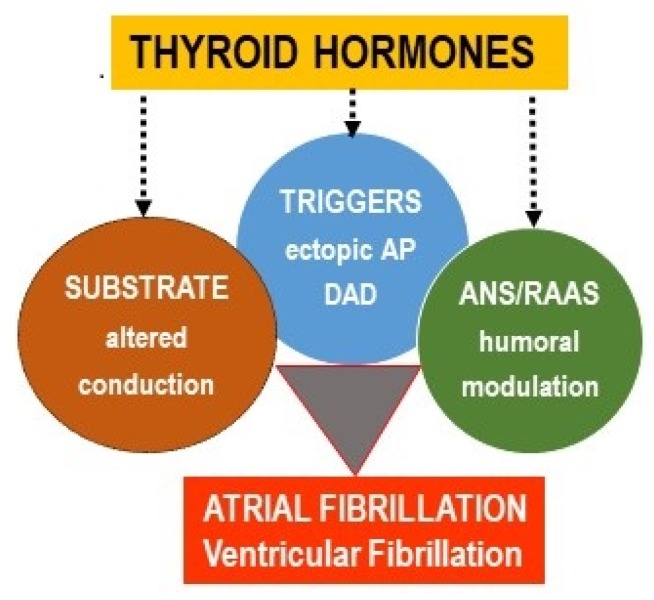
An excess of thyroid hormones (TH) promotes the occurrence of the main factors involved in cardiac arrhythmogenesis: Substrate, triggers, autonomous nervous system (ANS), and renin–angiotensin–aldosterone system (RAAS) imbalance.

Atrial fibrillation (AF) and ventricular fibrillation (VF) are clinically relevant and potentially life-threatening arrhythmias. Despite some differences, both AF and VF have been assumed to occur due to abnormalities in the electrical activity involved in impulse initiation and impulse propagation [1,2,3,4,5,6,7,8,9]. The former is due to an enhanced automaticity of the cardiomyocytes (i.e., pacemaker-like activity) or triggered activity expressed as early after-depolarization (EAD) or delayed after-depolarization (DAD). The latter is associated with a conduction block promoting a re-entrant excitation (most likely due to an electrical uncoupling), disorders at the intercellular connexin (Cx) channels, myocardial structural remodeling (hypertrophy and/or fibrosis, adiposity), and variations in the refractory periods. As mentioned in this review and depicted in Figure 1, thyroid hormones (TH) can be involved in all of these mechanisms, as well as in the modulation of the autonomous nervous system (ANS) and the renin–angiotensin–aldosterone system (RAAS) that affect arrhythmogenesis. 

The thyroid gland produces thyroxine (T_4_), which is a relatively inactive prohormone, and lower amounts of the active hormone, triiodothyronine (T_3_). About 20% of T_3_ is made by the thyroid gland and the other 80% comes from T_4_ upon conversion by type 1 iodothyronine deiodinases (IDs), which is widely distributed. Type 2 ID catalyzes the inactivation of FT_4_ and FT_3_ while type 3 ID acts like type 1 ID, and is expressed in skeletal and cardiac muscles, thus providing local intracellular production of T_3_ [10].

The release of TH from the thyroid gland is controlled by the thyrotrophin-releasing hormone from the hypothalamus in the brain and by the thyroid-stimulating hormone (TSH) produced by the pituitary gland. This forms part of a feedback loop known as the hypothalamic–pituitary–thyroid axis [11]. The thyroid gland can become overactive in hyperthyroidism or underactive in hypothyroidism. The latter is often accompanied by an enlargement of the thyroid gland known as goiter. Thyrotoxicosis may be the result of hyperthyroidism as in Graves’ disease (autoimmune hyperthyroidism), inflammation of the thyroid, benign thyroid tumor, or due to factors that affect hypothalamic, pituitary, or thyroid function [12]. Hyperthyroidism can also be caused by an acquired immune deficiency syndrome along with anti-retroviral therapy [13]. Moreover, a not-so-infrequent cause of hyperthyroidism in clinical practice is an iatrogenic one due to the adverse action of drugs, namely amiodarone. Apart from that, various gastrointestinal disorders may impede the absorption of T_4_ and increase the risk of developing iatrogenic hyperthyroidism [14].

Both overt and subclinical (latent) hyperthyroidism are endocrine disorders that occur as a result of excessive TH secretion. TSH is a sensitive indicator of thyroid function. Subclinical hyperthyroidism is common in the general population and its frequency is variable, depending on age, sex, and iodine status [15,16,17], and has been defined as lower serum TSH levels (<0.10 mlU/L), while the free T_4_ and T_3_ concentrations are within the reference range. The euthyroid status is defined as the condition wherein the TSH level is within the range from 0.45 to 4.49 mlU/L. In the National Health and Nutrition Examination Survey, 2.5% of the population had a serum TSH below the lower limit of the reference range [18].

TH affect heart metabolism, electrical properties, and function through the interplay of genomic and non-genomic mechanisms of action [17,19,20,21,22]. Accordingly, chronic and acute changes in the circulating TH have a fundamental impact on cardiac electrophysiology, Ca^2+^ handling, and structural remodeling. Disorders of these cellular factors due to a TH imbalance affect the susceptibility of the heart to arrhythmias [4,15,17].

Both overt and subclinical hyperthyroidism in humans increases the risk for cardiac arrhythmias, especially AF [17,23,24]. Recent data indicate that even in euthyroid individuals, the high-normal range of the circulating free T_4_ is associated with an increased AF incidence [25]. The incidence of VF attributed solely to the thyroid status imbalance is less frequent in humans, unlike the experimental animals, which are prone to both AF and VF in response to an excess of TH [15,26,27]. 

It should be noted that an exogenous T_4_ administration results in similar increases in circulation, as well as the T_3_ levels, in cardiac tissues [28]. Evidence suggests that thyroid dysfunction and thyroid diseases associated with a TH suppressive therapy may exert a clinically relevant impact on the susceptibility of the heart to arrhythmias, as well as the outcomes of treatment. This issue is even more complicated due to the fact that TH biosynthesis machinery has been detected in the heart and has been altered due to cardiac pathology [29].

## 2. Atrial Fibrillation and Pro-Arrhythmic Signaling of TH 

### 2.1. A Short Overview on AF

Concerning AF, the cardiomyocyte sleeves overlapping the pulmonary veins along with Ca^2+^ handling disorders are the sources of the ectopic electrical activity, while re-entry circuits are promoted by the atrial tissue heterogeneity and disorders in the intercellular electrical coupling mediated by connexin (Cx) channels [3,5,8,9,30,31,32,33,34]. The association of AF with the atrial Cx37 and Cx40 gene polymorphisms [35], as well as somatic mutations in *GJA5* (encoding Cx40), have been identified in AF [36,37]. As a pulmonary veins isolation-based approach can resolve AF in 50%–70% of patients, it implies that other drivers of AF remain to be determined [38]. TH may be one of those drivers for AF.

Risk factors for AF, such as aging, obstructive sleep apnea, diabetes, hypertension, dyslipidemia, obesity, cancer, renal dysfunction, and thyroid diseases, which are all accompanied by deleterious oxidative stress, may act synergistically to cause AF [5,15,39,40,41,42,43,44], whereby the noncoding microRNAs translate cellular stressors, such as reactive oxygen species, into AF pathogenesis [45]. Emerging evidence suggests a significant role of the altered atrial metabolism, phosphorylation of proteins, inflammatory and autoimmune channelopathies, and presence of autoantibodies to the M2-muscarinic and β 1-adrenergic receptors in the pathogenesis of AF [46,47,48,49,50,51,52,53,54]. Due to these mentioned chronic stressors implicated in electrical remodeling and poor risk factors control, the incidence of AF increases globally. 

AF, as recognized according to an irregular R–R interval and a missing P wave in an ECG, is a highly prevalent arrhythmia promoting heart failure, embolic stroke, and death [55]. Even short, subclinical episodes of AF are associated with an increased risk of stroke [56]. Paroxysmal, as well as sustained and permanent forms of AF, confer a significant clinical burden and worsens the patient’s quality of life. 

Management of AF includes antiarrhythmic drug therapies that are often ineffective in terminating AF or preventing its recurrences, possibly because these drugs target a single pathophysiological mechanism [8]. Catheter ablation of the arrhythmogenic triggers, another option of AF treatment, does not prevent recurrence of AF, probably because of the persistence of the arrhythmogenic substrate [55,57]. In the advanced form of AF, the abnormal atrial substrate, Cx43, Cx40, and Cx45 abnormalities are thought to act as drivers of arrhythmia perpetuation [35,41,58,59]. Modulation of the autonomic nervous system has shown promising alternatives to the standard AF treatment [60]. Nevertheless, a better understanding of the modifiable biomarkers, including an altered thyroid status, and molecular factors, including autoantibodies, may provide us with a chance to prevent AF or to tailor the treatment to avoid harmful consequences. It is noteworthy that women have worse and often atypical symptoms, as well as a higher risk for stroke and death, associated with AF compared to the risk in men [61].

It should, however, be emphasized that a considerable number of individuals have no AF symptoms [62,63], which is a major difficulty in an arrhythmia screening for detection of AF. Therefore, silent or subclinical AF is a major health concern, particularly because of its association with stroke. There is a need for novel approaches, as well as diagnostic and prognostic biomarkers [64]. Intermittent hand-held ECG recording revealed that the prevalence of AF in a population-based study was about 30% [65]. Patients with AF exhibit increased levels of the circulating N-terminal B-type natriuretic peptide (NT-proBNP), as well as the fibroblast growth factor-23 (FGF-23). Elevation of these markers can predict the development of AF in high-risk subjects or identify patients with AF [66,67,68]. In this context, it appears relevant to monitor TH status as well. 

### 2.2. Thyroid Status Imbalance Promoting AF

TH are one of the factors associated with AF and potential drivers of AF [16,22,69]. Increased automaticity and an enhanced triggered activity may increase the arrhythmogenic activity of cardiomyocytes at the pulmonary veins in hyperthyroidism [70]. The propensity for AF is increased in animals [71], as well as in humans with overt or subclinical hyperthyroidism that is more common in the general population and often accompanies various diseases [15,72,73]. Indeed, TSH levels ≤0.1 mIU/L have been linked with a three-fold increase in AF risk [17]. Even in euthyroid subjects with normal serum TSH levels, the free T_4_ concentration has been independently associated with AF [74]. Maximum P-wave duration and P-wave dispersion (indicators for the risk of paroxysmal AF) were longer in patients with endogenous and exogenous subclinical hyperthyroidism [72]. The incidence of paroxysmal AF is higher in toxic nodular goiter [75] and in patients suffering from Graves’ disease [76,77], which is caused by the presence of TSH autoantibodies directed toward the G protein-coupled TSH receptor [78]. The rate of intra- and extra-thyroidal conversion of T_4_ to T_3_ is elevated in Graves’ disease mostly due to an increased deiodase-1 activity. Thyroidectomy in Graves’ disease has been reported to abolish the AF load [77]. It is worthwhile to note that the occurrence of AF in this condition is associated with concomitant autoantibodies toward the β1-adrenergic and the M2 muscarinic receptors [79,80,81,82,83]. These autoantibodies and T_4_ facilitated the induction of AF in animal models as well [82]. Autoantibodies activating the G protein-coupled β1-adrenergic receptors in the heart have been implicated in the development of both AF and VF [84,85,86,87]. Thus, autonomic autoantibodies along with TH potentiate the vulnerability of the heart to AF. Considering the impact of TH on the development of AF, the screening for AF is highly relevant not only in patients with an overt but particularly with a subclinical hyperthyroidism. 

Moreover, TH affect the outcomes after an invasive treatment of AF. Accordingly, high circulating T_3_, as well as high-normal T_4_ or lower TSH levels, have been associated with AF recurrence after an arrhythmogenic foci ablation [23,88,89,90,91]. Free T_4_ levels influence the success rate of ablation procedures even in the normal range. Likewise, TH replacement therapy exerts an impact on the AF ablation outcome [92]. Despite sophisticated invasive approaches, the recurrence of AF remains an unresolved problem in clinical practice, challenging for further research [8]. 

## 3. Impact of Thyroid Hormones on Ventricular Arrhythmias

### 3.1. A Brief Overview on VF

Concerning VF, development of this life-threatening arrhythmia is similar to the development of a multifactorial AF [2,6,93]. VF is triggered by the dysfunction of ion and connexin channels along with abnormal Ca^2+^-handling and facilitated in the presence of an arrhythmogenic structural substrate (such as myocardial hypertrophy, fibrosis, and misdistribution of connexins). All these events are influenced by the modulating factors, such an ischemia or autonomous nervous system (ANS) and hormonal imbalance, including TH. Specific QRS complex patterns, recognized due to hypertrophy, may potentially predict ventricular arrhythmias [94]. When structural abnormalities are not evident, autoimmune channelopathies have been established as a novel mechanism in cardiac arrhythmias [51,52,53,54,95,96]. In particular, proarrhythmic autoantibodies targeting calcium, potassium, or sodium channels and anti-desmosome antibodies in the heart have been identified. These autoantibodies promote conduction disturbances and induce substantial electrophysiological changes facilitating life-threatening ventricular arrhythmias. 

Despite recognizing the basic mechanisms that can cause VF, the changes in the cardiac electrical properties remain poorly understood. Electrical disturbances result from the immediate operation of one or other arrhythmogenic mechanisms in different heart conditions. Accordingly, higher levels of total T_3_ have been positively associated with the heart rate, QTc, and negatively associated with the PR interval and QRS duration [97].

Notably, many pathophysiological processes implicated in the development of AF and VF are linked to a mitochondrial dysfunction, which causes an altered calcium homeostasis, an excess of reactive oxygen species formation (oxidative stress), and alterations in the oxygen consumption. Mitochondria are considered to be a metabolic sink and [98] the targets for suppressing arrhythmias [99].

Despite the progress in the treatment of heart diseases and the management of arrhythmias, sudden cardiac death, occurring due to malignant ventricular arrhythmias, remains a major cause of mortality globally [93]. An implantable cardioverter defibrillator may be efficient in preventing sudden death due to VF when it occurs but cannot prevent VF development and/or its recurrence. This issue remains to be investigated to reduce the risk of an incident VF.

### 3.2. Thyroid Status Imbalance Promotes VF

In contrast to the prevalence of AF, the incidence of VF attributed solely to the hyperthyroid status is less common and registered with a frequency similar to that in the euthyroid population [15]. It is likely because VF is exceptional in those cases where TH levels are elevated but without the observation of an arrhythmogenic structural substrate or channelopathies [100]. Nevertheless, ventricular tachycardia has been registered in hyperthyroid patients suffering from Grave’s disease and it has been associated with the interaction of autoantibodies of the β1-adrenergic, the M2 muscarinic, and the TSH-receptors [75]. While thyroidectomy in Graves’ disease attenuates the occurrence of ventricular arrhythmias [77], it seems likely that VF may occur in individuals with an altered thyroid status when accompanied by the presence of the autoantibodies. 

Autoimmunity may alter the myocardial electrical properties promoting idiopathic ventricular arrhythmias [101,102]. Recent data strongly point out the implication of autoantibodies toward the β1 and β2 adrenergic or the M2 muscarinic receptors and the myosin heavy chain in the occurrence of cardiac rhythm disturbances [80,84,86,87,103]. Anti-β adrenergic and anti-muscarinic receptor antibodies affect the myocardial electrophysiological properties and have been reported to be the independent predictors of sudden cardiac death in patients with various heart diseases [104]. The dysregulating effects of the autoantibodies against the calcium and potassium ion channels can play the basis for autoimmune phenocopies of genetic cardiac channelopathies [53]. Autoimmune cardiac channelopathies have been suggested as a novel mechanism in the development of cardiac arrhythmias [52].

Occasionally, acute thyrotoxicosis accompanied by severe hypokalemia can induce a persistent ventricular tachycardia [105]. Unlike poor evidence in humans, the impact of TH on the development of VF in experimental animals is well documented [26,106,107,108] and associated with both the genomic and non-genomic TH actions [109]. Perhaps because of high TH dose, the oxidative stress-related impairment of ion and Cx43 channels is more pronounced in animal models.

Concerning TH, the prevalence of atrial versus ventricular arrhythmias may be explained by the chamber-related differences in the expression of ion and connexin channels, the duration of the effective refractory period, conduction velocity, and local activation time [15,110,111], as well as in numerous signaling pathways [112] and distinct tissue pro-fibrotic properties [32]. Certainly, hyperthyroidism promotes a myocardial electrical instability [113] due to an increased excitability and shortening of the repolarization, thereby facilitating triggered activity and ventricular premature beats that often initiate malignant arrhythmias in a structurally altered heart [114]. TH may also affect ventricular arrhythmogenesis via an influence of the ANS [115] as the density of the adrenergic binding sites has been shown to be enhanced by a chronic or an acute treatment with TH [116,117].

## 4. Thyroid Malignancies, Treatment, and Risk for Arrhythmias 

### 4.1. Pathomechanisms, Incidence, and Treatment of Thyroid Carcinoma (TCA)

Thyroid dysfunction and diseases are more prevalent in women (about 5–20 times higher than those in men) and their incidence is more pronounced with age [118]. Likewise, the incidence of differentiated thyroid cancer (DTCA) increases in the population over 55 years [119]. Papillary and follicular thyroid carcinoma are the most common types of DTCA [120]. Undifferentiated medullary and anaplastic thyroid cancer have, however, a less favorable prognosis than that for DTCA ([121]. 

Inflammation and oxidative stress are involved in the pathogenesis of cancer in a manner similar to that in cardiovascular diseases [122,123,124,125] by contributing to the initiation, progression, and complication of these diseases [126]. The genetic landscape is clearly evident and gene rearrangements are classified according to a spectrum of the RAS-like and BRAF-like tumors [127]. BRAF^V600*E*^ mutation positivity and mutations in the telomerase reverse transcriptase promoter may also be prognostic in TCA progression or recurrence [128,129]. A mutational analysis helps to define the appropriate initial management, adjuvant therapy, surveillance protocols, and treatment [130]. 

Standard management of the TCA patients includes a total thyroidectomy, a neck lymph node dissection (if indicated), and radioactive iodine (RAI) ablation followed by a TSH suppressive T_4_ therapy. Surgery is the primary crucial treatment to remove the tumor and the involved regional lymph nodes, to facilitate an accurate staging of the disease, to minimize the risk of disease recurrence and metastasis, and to facilitate a postoperative treatment with RAI and an accurate long-term surveillance for recurrence [131]. The accuracy of the post-radioiodine SPECT/CT predicts a long-term outcome of DTCA [128,132].

Total thyroidectomy followed by RAI has been associated with benefits in high-risk patients, as well as in the decrease in TCA recurrence. Current guidelines recommend an individualized approach for RAI indication [133] because its benefit in low-risk patients was not supported by evidence [134,135,136]. Despite the potential adverse effects of RAI, its application increases in patients with DTCA [137,138]. Decreased quality of life in TCA survivors has been recently reported [139], but there is a lack of information about arrhythmias. 

Post-surgery suppressive therapy with T_4_ downregulates the pituitary TSH that promotes growth of the residual malignant thyroid cells [140]. Thus, lowering the TSH levels with an exogenous T_4_ is involved in the long-term management of DTCA [141]. The benefit of this therapy has been established for high-risk but not for low-risk patients [134]. Long-term treatment with T_4_ affects the TH metabolism and results in a stable subclinical hyperthyroid state along with the down-regulation of deiodinases 1–2 and upregulation of deiodinase 3 [142]. Whether these patients exhibit cardiac rhythm disturbances is not known, while an inverse correlation between TSH levels and the risk of AF has been well established in a benign thyroid disease [143].

Besides exogenous T_4_, TH analogs possessing a thyroidomimetic activity, such as triiodothyroacetic acid (an acetic acid metabolite of T_3_) and tetraiodothyroacetic acid (a derivate of T_4_), reduce the risk of cancer progression, enhance therapeutic effects, and suppress cancer recurrence [144]. However, all exogenous approaches exert adverse cardiovascular effects as indicated by earlier and recent studies [140,145,146,147,148,149]. It is challenging for cardiac arrhythmia screening. 

The thyroid axis ‘set-points’ are significantly altered after a long-term T_4_ therapy and the dosage required for the TSH suppression may be reduced over time [150]. However, the appropriate degree of TSH suppression remains unsettled according to discrepancies among guidelines [151]. Nevertheless, TSH in the low-normal range <1 mIU/L seems to be preferable in most patients with DTCA [133]. In the absence of prospective trials, the discussion still remains concerning the extent of surgery, benefit and dosing of postoperative RAI, as well as the optimal level and duration of a TH-suppressive therapy [134]. The updated trials should also pay attention to arrhythmia risk stratification when considering predisposition to AF, including asymptomatic.

### 4.2. Thyroid Carcinoma Suppressive Therapy and Risk for Cardiac Arrhythmias

There is a link between AF and thyroid nodules, whereby cytokines and growth factors, such as IGF-1, EGF, and FGF, may be involved [152]. Moreover, TH regulate oxidative metabolism and plays an important role in the production of free oxygen radicals, thereby promoting oxidative stress. This event, as well as autoimmune thyroiditis, has been shown to be involved in the pathogenesis, as well as adverse effects in patients suffering from TCA [122,123,124,125]. Elderly persons, even clinically euthyroid but with low or low-normal TSH level, are at increased risk for AF [153].

TCA is considered an independent predictor for AF along with well-known AF risk factors [140,154,155]. Notably, the increased risk of AF in patients treated for DTCA but without pre-existing cardiovascular disease may warrant periodic screening for this arrhythmia [156]. This is in line with a recent study indicating that patients with DTCA along with TSH suppression below 0.1 mIU/L have a higher risk of AF [151]. Some trials did not find an association between the TSH level but have found an association between the cumulative dose of RAI and AF within the TCA cohort [140]. 

**Figure 2:**
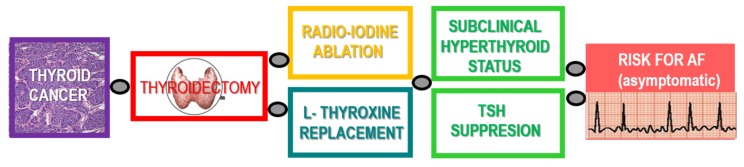
Algorithm of the thyroid cancer therapy and possible risk for development of atrial fibrillation (AF) due to subclinical hyperthyroidism.

According to a Swedish Nationwide Study, DTCA patients have a higher AF incidence compared to the general population, and females face a slightly higher incidence of cerebrovascular disease [157]. Thus, it appears relevant to pay attention to the AF risk factors as well as to an early identification of cardiac rhythm disturbances in TCA patients during their follow up (Figure 2). It may prevent AF-related complications and reduce the symptoms. The need for accurate risk stratification and long-term outcome data to support the treatment decisions is highly relevant for those with early-stage, very low-risk tumors, as well as for those with advanced and metastatic disease [134]. There is also limited information regarding the DTCA treatment-related morbidity associated with a long-term hypocalcemia [158] that may provoke rhythm disturbances. 

In the context of arrhythmias, an interesting question arises as to whether TCA patients during follow-up exhibit some specific risk factors for cardiac arrhythmias, such as the pro-arrhythmic β1-adrenergic and M2 muscarinic receptor autoantibodies or changes in the myocardial connexins. It has been shown that TSH and TH regulate the expression of connexins in a variety of target tissues, including the thyroid gland [159]. Reduced connexin-43 expression in the thyroid tissue differentiates TCA from a benign disease and provides clinical utility as a marker for malignancy [160,161], while decreased connexin-43 protein abundance in the cardiac tissue in the experimental hyperthyroid setting increases the propensity to AF and VF [26,27,108,162]. On the other hand, the upregulation of atrial connexin-40 precedes AF as well [163]. Alteration of both connexin-43 and connexin-40 abundance impairs an intercellular electrical coupling, thereby affecting the myocardial conduction and facilitating cardiac arrhythmias [5,6].

## 5. Cellular and Molecular Actions Potentially Involved in Pro-Arrhythmic Signaling of TH

TH are powerful modulators of electrical properties of the heart [15,17,23,164], thus playing an important role in the development of cardiac arrhythmias that occur due to electrical disturbances. Molecular targets for TH implicated in electrical instability that may facilitate AF or VF occurrence have been summarized in Table 1. 

### 5.1. Targeting Cardiac Ion Channels 

Microarrays analyses showed that the hyperthyroid status increased the expression levels of genes encoding the voltage-gated potassium channel proteins, notably *KCNA5* (Kv1.5) and *KCNB1* (Kv2.1), that contributed to the main repolarizing K^+^ currents, as well as the *HCN2* and *HCN4* genes encoding pacemaker *I*_f_ channels [178]. In contrast, the expression of *KCNQ1* (KvLQT1) and its regulator *KCNE1* (minK) generating the slow component of cardiac delayed rectification, *I*_Ks_ current, was decreased. The up-regulation of Kv1.5 mRNA was greater in the atrium than that in the ventricle.

Electrophysiological studies revealed that the sinus tachycardia was related to the effect of TH on the rate of diastolic depolarization of the pacemaker cells via the increase in the pacemaker HCN2 (*I*_f_) current [165,166]. In this context, it should be noted that the hyperpolarization-activated cyclic nucleotide-gated HCN channels isoforms are also expressed in the atrial and ventricular tissue; therefore, they may be implicated in promoting a triggered activity in a pathophysiological setting [179]. Indeed, TH can activate electrical triggers inducing an abnormal depolarization [180] that often arises from the cardiomyocytes overlapping the pulmonary veins. 

Enhanced triggered activity may increase the arrhythmogenic activity due to a higher incidence of DAD that often initiate AF [70]. Delayed-rectifier *I*_Kur_ and acetylcholine-regulated *I*_KAch_ currents are present solely in the atrial tissue [181]. The shortening of the action potential duration (APD) in hyperthyroid rat atria was linked with a remarkably increased *I*_Kur_ and a decreased L-type Ca^2+^ current [169,173,182], while the latter was increased in the ventricle [172]. Th shortening of the APD associated with decreased refractoriness has been considered as one of the main mechanisms for the risk of AF in hyperthyroidism [166,173,183]. These conditions likely affect the P-wave duration and may increase the P-wave dispersion predisposing to an AF [184].

It is noteworthy that the interaction of β1-adrenergic receptor autoantibodies with the target receptor amplifies the L-type Ca^2+^ current (*I*_Ca_) and rapid delayed rectifier K^+^ currents (*I*_ks_, *I*_kr_), which result in a decreased APD and the increase in the membrane potential during the plateau phase [53]. Interaction of the M2 muscarinic receptor autoantibodies with the target receptor inhibits ICa and increases the outward acetylcholine-regulated potassium current (*I*_K,Ach_), which results in a hyperpolarization and shortening of APD [85].

### 5.2. Targeting Cardiac Ca^2+^ Handling

Thyroid status affects myocardial Ca^2+^ handling and intracellular free Ca^2+^ concentration by regulating the transcription of the genes involved in Ca^2+^ cycling [11]. Both gene expression and protein levels of sarcoplasmic reticulum Ca^2+^-ATPase (SERCA2a) increase in response to TH [15], along with suppression of phospholamban expression [169]. It suggests an increase in Ca^2+^ uptake by sarcoplasmic reticulum whereby its Ca^2+^ overload may facilitate spontaneous Ca^2+^ leaks. An abnormal Ca^2+^ cycling and Ca^2+^ leak through the ryanodine receptor (RyR) in sarcoplasmic reticulum are involved in the occurrence of triggered activity such as EAD or DAD, followed by premature contraction [170]. DAD may occur during tachycardia in hyperthyroidism and contribute directly to increased susceptibility of the heart to AF and VF [8]. Moreover, TH increased the *I_N_*_a_ current, resulting in an increase in intracellular Ca^2+^ likely via an accelerated reverse mode of Na^+^/Ca^2+^ exchanger [167]. High diastolic Ca^2+^ and Ca^2+^ overloads inhibit intercellular communication mediated by connexin channels that may result in a slower or block of conduction promoting re-entrant arrhythmias [171,174].

### 5.3. Targeting Myocardial Metabolism, Structure, and Intercellular Coupling 

TH induce a hyperdynamic cardiovascular state accompanied by an elevation of the left atrial pressure and increase in the size of the atria that facilitates a paroxysmal AF [185]. It suggests that, apart from the ion and Ca^2+^-handling alterations, AF occurrence is promoted by the presence of a structural arrhythmogenic substrate, such as hypertrophy. This is linked with alterations in the expression, phosphorylation, and distribution of the Cx40, Cx43, and Cx45 channels [35,41,58,59,162,163]. Impairment of the connexin channels deteriorates the electrical coupling required for an AP propagation, thereby affecting the myocardial conduction and promoting re-entry mechanisms [8]. Hyperthyroid rats exhibited a down-regulation of Cx43 in both the atrial and ventricular tissues and were prone to an electrically inducible AF and VF [21,101,158]. In this context, it should be noted that functional phosphorylated forms of Cx43 were markedly suppressed in both the atria and ventricles of hyperthyroid rats. It can be attributed to the down-regulation of the protein kinase C epsilon (PKCε) that is suppressed by TH [15,20]. 

Moreover, a cardiac overload of various etiologies (including hyperthyroidism) is associated with an altered myocardial redox state and oxidative stress, which impairs the function of numerous target proteins including the ion and connexin channels, thereby facilitating the development of AF or VF [5,6].

### 5.4. Targeting ANS and RAAS 

TH interact with the sympathetic nervous system by augmenting responsiveness to a sympathetic stimulation presumably via modulating the adrenergic receptor function and/or density. Myocardial adrenergic receptor binding sites have been shown to be enhanced by a chronic, as well as an acute treatment with TH [15]. There is also cross-talk between TH and the renin–angiotensin–aldosterone system [186]. TH activate RAAS through the TH response elements (TREs), thereby increasing the expression of mRNA for renin. Taken together, it seems realistic that ANS and RAAS may be additional players implicated in the development of arrhythmias in hyperthyroidism. 

## 6. Conclusions

TH regulate multiple nuclear and extra-nuclear processes that operate to maintain the cardiac function. An overt or more frequent subclinical hyperthyroid state due to thyroid diseases disturb this regulation, promoting the development of cardiac arrhythmias, mostly AF. Disorders in the intracellular Ca^2+^ due to an altered Ca^2+^ handling, along with alteration of the expression and function of the HCN, Na^+^, K^+^, and Ca^2+^ channel and an impairment in the connexin channel-mediated cell-to-cell coupling, seem to be crucial factors in the proarrhythmic signaling of TH. Accordingly, it appears that the long-term subclinical hyperthyroid state including a TSH suppressive therapy with L-thyroxine may increase the risk for AF in post-thyroidectomy patients. Though relatively uncommon, perhaps because of a lack of real evidence due to limitations in the screening process [187], awareness about potential interactions promoting AF, particularly asymptomatic, and to prevent undesirable consequences is required.

## Figures and Tables

**Table 1:** Cellular actions potentially involved in pro-arrhythmic signaling of TH in the heart.

Target Molecule	Action	Response/Putative Effects
**Nuclear TH receptor-mediated actions**		
HCN2 channels	*Upregulation* [165,166]	Enhanced pacemaker activity
Na^+^ K^+^ ATPase	*Upregulation* [167]	Hyperpolarization/Enhanced excitability
Ca^2+^ ATPase	*Upregulation* [168]	Altered Ca^2+^ handling/Triggered activity
Ryanodine receptor	*Upregulation* [106]	Spontaneous Ca^2+^ leak/Triggered activity
Kv1.5; 4.2; 4.3 channels	*Upregulation* [169]	K^+^ current increase/Shortening APD
Connexin-40	*Upregulation* [163]	Altered intercellular electrical coupling
β1-adrenergic receptor	*Upregulation* [170]	Sympathetic overdrive
α-myosin heavy chain	*Upregulation* [171]	Structural remodeling/Enhanced contractility
Phospholamban	*Downregulation* [168]	Altered Ca^2+^ handling/Triggered activity
Na/Ca exchanger	*Downregulation* [168]	Altered Ca^2+^ handling/Ca^2+^ overload
L-Ca^2+^ channels	*Downregulation* [172]	Altered Ca^2+^ handling/Triggered activity
Kv 1.2; Kv 1.4 channels	*Downregulation* [169,173]	K^+^ current increase/Shortening APD
Connexin-43	*Downregulation* [107]	Altered intercellular electrical coupling
α1-adrenergic receptor	*Downregulation* [116]	Sympathetic overdrive
Protein kinase C-ε	*Downregulation* [174]	Reduced protein (Cx) phosphorylation
β-myosin heavy chain	*Downregulation* [171]	Structural remodeling/Enhanced contractility
**Non-nuclear receptor-mediated actions of TH**		
HCN2 (I_f_) current	*Activation* [165,166]	Enhanced pacemaker activity
Ca^2+^ ATPase	*Activation* [172]	Altered Ca^2+^ handling
Na^+^ K^+^ ATPase	*Activation* [21,167]	Hyperpolarization/Enhanced excitability
L-Ca^2+^ channels	*Suppression* [172]	Altered Ca^2+^ handling
Ryanodine channels	*Activation* [175]	Altered Ca^2+^ handling
Na+ channels	*Activation* [176,177]	Hyperpolarization
Na/Ca exchanger	*Activation* [21]	Altered Ca^2+^ handling
K^+^ channels	*Activation* [21,177]	Shortening APD
β-adrenergic receptors	*Activation* [117]	Sympathetic overdrive
